# Pain, Side Effects, and Abortion Experience Among People Seeking Abortion Care in the Second Trimester

**DOI:** 10.1089/whr.2021.0103

**Published:** 2022-05-12

**Authors:** Ilana G. Dzuba, Sruthi Chandrasekaran, Laura Fix, Kelly Blanchard, Erin King

**Affiliations:** ^1^ Consultant, New York, NY, USA.; ^2^ Ibis Reproductive Health, Cambridge, Massachusetts, USA.; ^3^ Hope Clinic for Women, Granite City, Illinois, USA.

**Keywords:** second-trimester abortion, pain, side effects, abortion experience, gestational age, secondary analysis

## Abstract

**Background:**

There is limited documentation about pain and side effects associated with dilation and evacuation (D&E) abortion, yet, pain and side effects are important factors that can affect a client's abortion experience. In 2016, Hope Clinic for Women, an independent abortion clinic in Illinois, altered its cervical preparation protocols before D&E to reduce the total time of the abortion process and improve the client experience. This analysis addresses the gap in data on client experience of abortion in the later second trimester by evaluating pain, side effects, and acceptability by gestational age.

**Methods:**

Abortion clients obtaining services at the clinic between March 2017 and June 2018 were eligible to participate if they had viable singleton pregnancies of 16–23.6 weeks' gestation, spoke English, and were at least 18 years old. Eligible participants completed a two-part survey about their abortion experience.

**Results:**

We found that respondents seeking abortion care at later gestations in the second trimester were more likely to report pain during their abortions. We did not find any association between side effects and gestational age.

**Conclusion:**

Although most respondents were prepared for the pain they experienced, some reported experiencing more pain than they expected, and more effective pain relief was commonly reported as a way to improve the service. More research on patient experiences of later abortion is needed, particularly on experiences of pain and options for pain management.

## Introduction

Approximately 5.4% of facility-based abortion procedures in the United States in 2017 were performed among people seeking care with pregnancies at 16 or more weeks of gestation.^1^ Dilation and evacuation (D&E) is the method most commonly used in this country for mid to late second-trimester abortion and is very safe and effective.^2^ Achieving adequate cervical dilation is an important factor in avoiding potential complications with the procedure, such as hemorrhage and cervical laceration.^3,4^

In 2016, Hope Clinic for Women, an independent abortion clinic in Illinois, altered its cervical preparation protocols before D&E to reduce the total time of the abortion process and improve the client experience.^5–12^

For pregnancies 16–17.6 weeks of gestation, osmotic dilators were discontinued and replaced with a 400 μg misoprostol (a widely available, heat stable, inexpensive E1 prostaglandin analog) dose 120 minutes before D&E, which studies show that clients generally prefer because of discomfort from dilator placement and shorter procedure duration,^10,12,13^ despite the greater likelihood of need for additional mechanical dilation.^14–16^

For pregnancies 21–23.6 weeks, mifepristone 200 mg (an antiprogestin) was added as an adjunct to osmotic dilators on day 1, which providers report may make D&E procedures easier.^11^ On day 2, if dilation was adequate, misoprostol 400 μg was given 0–180 minutes before D&E. If dilation was determined to be insufficient by the provider, a second set of osmotic dilators were administered and the D&E was completed on day 3.

The protocol for pregnancies 18–20.6 weeks did not change—osmotic dilators are placed day 1, and on day 2, the D&E procedure was completed 0–180 minutes after misoprostol 400 μg.

These protocols are in line with the Society of Family Planning's recommendations for cervical preparation before second trimester abortion up to 24 weeks gestational age.^17,18^ Intrafetal or intra-amniotic injection of 500 μg to 1.5 mg of digoxin was administered to everyone with pregnancies of 18 weeks and more.

There is limited documentation about pain and side effects associated with D&E abortion, yet, pain and side effects are important factors that can affect a client's abortion experience.^19,20^ People who undergo D&E procedures report pain with dilator insertion, cervical dilation, injections to administer paracervical block and digoxin, uterine contractions, and postprocedure recovery.^9,11,13,15,16^ Digoxin is associated with vomiting,^21^ and common side effects of misoprostol, if used for cervical preparation, include gastrointestinal upset as well as fever and chills. Misoprostol-associated side effects are transient and can be managed with gastrointestinal treatments such as antiemetics and antidiarrheals. Pain and side effect management, therefore, is a meaningful component of quality of care.^22^

Currently, there is no standardized approach nor clear recommendations for pain management during later abortion procedures.^23^ Given the limited data, Hope Clinic aimed to reduce overall pain and improve client experience by reducing the need for a second set of osmotic dilators.^5^

At Hope Clinic, a combination of preoperative oral analgesia with ibuprofen, anxiolysis with alprazolam, and intraoperative moderate sedation with fentanyl and versed was used for pain management during the D&E procedure. Oral analgesia with ibuprofen alone was administered for osmotic dilator insertion. Patients were given the oral analgesia medications such as ibuprofen and an acetaminophen combination with opioid at discharge on day 1 for self-administered use throughout their abortion process outside of the facility.

Other intravenous or intramuscular analgesia medications such as ketorolac and antiemetic medications such as ondansetron were available as needed for in-facility side effect management. Verbal anesthesia, heat, and uterine massage were also used for pain management. The pain management strategies in use at Hope Clinic reflect Society of Family Planning recommendations for using multiple interventions to address pain and improve patient experience.^24^ Pain and side effect management did not change with the altered cervical preparation protocol.

The D&E procedure is used across a broad gestational age range in the second trimester, but client experiences with pain and side effects may vary within that range based on the duration of the pregnancy. This analysis attempts to address the information gap in client experience of abortion in the later second trimester by analyzing data on pain, side effects, and acceptability by gestational age. A better understanding of client experiences among different gestational age cohorts will highlight potential ways to offer a higher quality and client-centered approach to abortion care.

## Materials and Methods

We conducted an analysis on pain, acceptability, and side effects using data collected in a cross-sectional self-administered survey of later abortion clients at Hope Clinic with pregnancies of 16–23.6 weeks to compare the experiences of people who underwent original and modified cervical preparation protocols before D&E procedures. This article explores self-reported pain, side effects, and acceptability of the D&E procedure by gestational age group (16–17.6, 18–20.6, 21–23.6 weeks). The study was approved by Allendale Institutional Review Board.

Abortion clients obtaining services at the clinic between March 2017 and June 2018 were eligible to participate if they had viable singleton pregnancies of 16–23.6 weeks' gestation, spoke English, and were at least 18 years old. Clinic staff did not recruit clients in emotional distress or those reporting safety concerns.

Clients learned about the study from a printed information sheet and/or verbally from clinic staff after undergoing abdominal ultrasound to confirm intrauterine pregnancy and estimate gestational age before initiating their abortions. Those interested in participating were given a packet with study information and informed consent procedures, a paper version of the self-administered, structured anonymous survey, and instructions on how to respond to the survey online (Qualtrics, Provo, UT) with a unique personalized link on a tablet at the clinic or on a personal device. Participants could instead opt to complete the paper survey and return in a preaddressed and stamped envelope.

Clients consented to participating in the study before proceeding to the survey questions. Those respondents who opted to use a paper survey could submit the completed survey to clinic staff in-person or in a self-addressed stamped envelope included in the packet.

The survey was completed in two sections. Respondents filled out the first section of 37 questions, including demographic information, before initiating their abortion procedures. Participants responded to an additional 32 questions postprocedure about the experience with the abortion procedure, including pain, side effects, and acceptability, as well as about clinic interactions. Participants who completed the first part of the survey received a $5 gift card and those who completed the remainder of the survey received an additional $20 gift card. Participants who completed the entire survey were entered into a raffle for one additional $100 gift card. The data used for this analysis were from the postprocedure portion of the survey.

Respondents rated the pain they experienced at different moments of the abortion process: cervical preparation in the clinic on day 1, cervical preparation after leaving the clinic on day 1, cervical preparation in the clinic on day 2 (if applicable), cervical preparation after leaving the clinic on day 2 (if applicable), digoxin administration on procedure day (if applicable), and the overall abortion process. They selected a number from 1 (no pain) to 10 (worst pain possible) on a visual analog pain scale, with corresponding circles of increasing size, that best reflected their pain.

Respondents also classified their perceptions about actual versus expected pain, actual versus expected side effects, actual versus expected reaction to abortion experience, the overall abortion experience, and care received at the clinic by Likert scales, and they were invited to offer recommendations to improve the abortion process.

For this analysis, we grouped all data by gestational age cohort (16–17.6, 18–20.6, and 21 weeks and beyond), regardless of cervical preparation protocol used. Data were analyzed with SPSS 20 (IBM, Armonk, NY), with *p* < 0.05 considered significant. We used Pearson's chi square and Fisher's exact test as appropriate to evaluate group differences for outcomes based on categorical variables, the Kruskal-Wallis nonparametric test for multiple comparisons, and the Dunn test with Bonferroni correction as appropriate for *post hoc* adjusted pairwise comparisons.

## Results

**FIG. 1.**
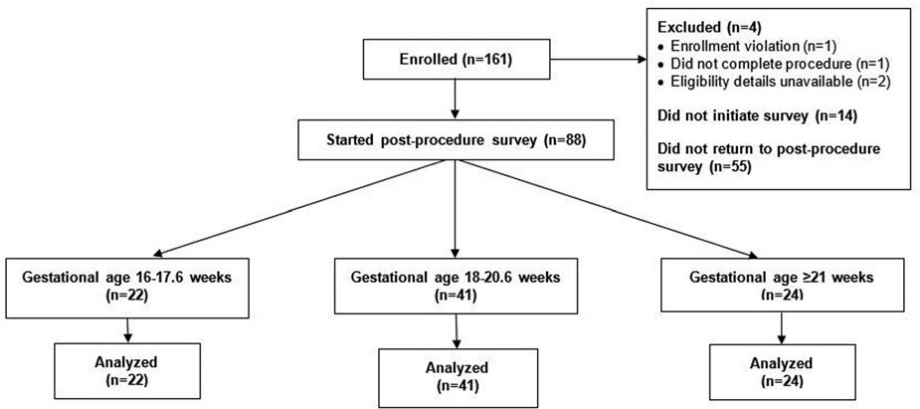
Flow of survey participants.

A total of 161 partcipants enrolled in the study. Two participants were excluded due to ineligibility: one was pregnant with a multiple gestation and another decided not to continue with the abortion procedure. Two additional were enrolled but eligibility details were unavailable and hence were excluded. Fifty-five (38.5%) of the 143 included respondents who initiated a survey did not return to the survey postprocedure. A total of 88 respondents who responded to the postprocedure survey portion were included in this analysis: 22 with pregnancies of 16–17.6 weeks, 42 with pregnancies of 18–20.6 weeks, and 24 with pregnancies 21–23.6 weeks (Figure 1).

Enrolled participants ranged from 18 to 42 years old; median pregnancy duration was 19 (interquartile range 17–21) weeks. Respondents identified primarily as black or African American (60.0%), followed by white (30.6%), Asian/Native Hawaiian or other Pacific Islander (2.4%), or other/mixed race (7.1%). No one in the sample identified ethnically as Hispanic, Latin, or Spanish, while 6.0% reported other ethnicity without specifying. Participants with pregnancies 21–23.6 weeks were more likely to have private insurance than those with pregnancies of <21 weeks. They were also more likely to be pregnant for the first time and be nulliparous. Table 1 shows the demographic characteristics of the surveyed population by gestational age cohort. The respondents who completed the entire survey were not sociodemographically different from those who responded to the first portion of the survey only (data not shown).

**Table 1.** Demographic Characteristics of Respondents by Gestational Age (Weeks)

	16–17.6	18–20.6	≥21	Total	*p* ^a^
Gestational age	*N* = 22	*N* = 41	*N* = 24	*N* = 87	
Median Gestational age in weeks (IQR)	16 (16–17)	19 (18–20)	23 (22–23)	19 (17–21)	<0.001
Age	*N* = 22	*N* = 41	*N* = 24	*N* = 87	
Median age in years (IQR)	26 (22–29)	26 (22–31.5)	24.5 (21–29)	25 (22–30)	0.64
Race, % (*n*)	*N* = 22	*N* = 39	*N* = 24	*N* = 85	0.18
Asian/Native Hawaiian Pacific Islander	0 (0)	0 (0)	8.3 (2)	2.4 (2)	
Black or African American	68.2 (15)	66.7 (26)	41.7 (10)	60.0 (51)	
White	27.3 (6)	28.2 (11)	37.5 (9)	30.6 (26)	
Other	4.5 (1)	5.1 (2)	12.5 (3)	7.1 (6)	
Ethnicity, % (*n*)	*N* = 22	*N* = 38	*N* = 24	*N* = 84	0.79
Non-Hispanic	95.5 (21)	92.1 (35)	95.8 (23)	94.0 (79)	
Other (nonspecified)	4.5 (1)	7.9 (3)	4.2 (1)	6.0 (5)	
Highest level of education, % (*n*)	*N* = 22	*N* = 39	*N* = 24	*N* = 85	0.11
Some or all of high school	72.7 (16)	84.6 (33)	54.2 (13)	72.9 (62)	
Associate/Bachelor's Degree	22.7 (5)	12.8 (5)	33.3 (8)	21.2 (18)	
Master's Degree/Other	4.5 (1)	2.6 (1)	12.5 (3)	5.9 (5)	
Household income, % (*n*)	*N* = 20	*N* = 33	*N* = 23	*N* = 76	0.08
Less than $15,000	60.0 (12)	54.5 (18)	34.8 (8)	50.0 (38)	
$15,000–$34,999	40.0 (8)	36.4 (12)	34.8 (8)	36.8 (28)	
$35,000–$49,999	0 (0)	6.1 (2)	8.7 (2)	5.3 (4)	
$50,000 and over	0 (0)	3.0 (1)	21.7 (5)	7.9 (6)	
Health insurance, % (*n*)^b^	*N* = 22	*N* = 40	*N* = 24	*N* = 86	
Private	13.6 (3)	7.5 (3)	45.8 (11)	19.8 (17)	0.001
Medicaid	50.0 (11)	52.5 (21)	29.2 (7)	45.3 (39)	0.17
Medicare	9.1 (2)	2.5 (1)	8.3 (2)	5.8 (5)	0.47
None	27.3 (6)	30.0 (12)	8.3 (2)	23.3 (20)	0.12
Unsure	4.5 (1)	2.5 (1)	8.3 (2)	4.7 (4)	0.56
Other	0 (0)	5.0 (2)	0 (0)	2.3 (2)	0.31
Working or in school, % (*n*)^b^	*N* = 22	*N* = 40	*N* = 24	*N* = 86	0.29
Working full-/part-time	31.8 (7)	47.5 (19)	54.2 (13)	45.3 (39)	
Student/unemployed	68.2 (15)	52.5 (21)	45.8 (11)	54.7 (47)	
Gravidity	*N* = 22	*N* = 39	*N* = 24	*N* = 85	
Primigravid, % (*n*)	9.1 (2)	15.4 (6)	45.8 (11)	22.4 (19)	0.004
Median gravidity	4 (2.75–5)	2 (2–4)	2 (1–2.75)	2 (2–4)	0.002^c^
Parity	*N* = 22	*N* = 40	*N* = 23	*N* = 85	
Nulliparous, % (*n*)	9.1 (2)	30.0 (12)	47.8 (11)	29.4 (25)	0.03
Median parity	2 (1–2)	1 (0–2)	1 (0–1)	1 (0–2)	0.012^d^
Stillbirths	*N* = 21	*N* = 39	*N* = 24	*N* = 84	
% (*n*)	0 (0)	2.6 (1)	4.2 (1)	2.4 (2)	0.66
Median stillbirths	0 (0–0)	0 (0–0)	0 (0–0)	0 (0–0)	0.65
Adopted children	*N* = 21	*N* = 39	*N* = 24	*N* = 84	
% (*n*)	0 (0)	2.6 (1)	0 (0)	1.2 (1)	0.56
Median adopted children (IQR)	0 (0–0)	0 (0–0)	0 (0–0)	0 (0–0)	0.57
Miscarriages	*N* = 22	*N* = 40	*N* = 24	*N* = 86	
% (*n*)	27.3 (6)	22.5 (9)	8.3 (2)	19.8 (17)	0.23
Median miscarriages	0 (0–1)	0 (0–0)	0 (0–0)	0 (0–0)	0.26
Previous abortions	*N* = 22	*N* = 40	*N* = 24	*N* = 86	
% (*n*)	54.5 (12)	50.0 (20)	25.0 (6)	44.2 (38)	0.08
Median previous abortions	1 (0–1)	0.5 (0–1)	0 (0–0.75)	0 (0–1)	0.15
Children in household	*N* = 22	*N* = 40	*N* = 24	*N* = 86	
% (*n*)	81.8 (18)	72.5 (29)	54.2 (13)	69.8 (60)	0.11
Median children in household	1.5 (1–2)	1 (0–2)	1 (0–1)	1 (0–2)	0.08

Includes participants who partially completed postprocedure survey.

^a^ Pearson chi square to compare proportions; Kruskal-Wallis to compare medians.

^b^ Respondents could select more than one response.

^c^ Dunn-Bonferroni adjusted pairwise comparison between 16–17.6 and ≥21 weeks: *p* = 0.002.

^d^ Dunn-Bonferroni adjusted pairwise comparison between 16–17.6 and ≥21 weeks: *p* = 0.009.

IQR, interquartile range.

### Pain

Nearly all (94.2%) respondents reported pain at some point during the abortion process, regardless of gestational age. Pain differed by gestational age after day 1, on day 2, after day 2, and overall. All respondents in the 21–23.6 weeks group reported pain on day 2, after day 2, and overall (Table 2).

**Table 2.** Reported Pain and Pain Scores at Different Moments in Abortion Process by Gestational Age (Weeks)

	16–17.6	18–20.6	≥21	Total	*p* ^a^
Cervical prep on first day	*n* = 16	*n* = 41	*n* = 23	*n* = 80	
Reported pain, % (*n*)	81.2 (13)	82.9 (34)	91.3 (21)	85.0 (68)	0.60
Median pain score scale 1–10 (IQR)	4 (2.25–6.5)	7 (3–8)	5 (3–8)	5 (3–8)	0.29
Cervical prep after going home first day	*n* = 21	*n* = 40	*n* = 24	*n* = 85	
Reported pain, % (*n*)	61.9 (13)	85.0 (34)	91.7 (22)	81.2 (69)	0.03
Median pain score scale 1–10 (IQR)	3 (1–4)	5 (2.25–7.75)	3 (2–5.75)	4 (2–6)	0.003^b^
Cervical prep on second day	*n* = 9	*n* = 29	*n* = 21	*n* = 59	
Reported pain, % (*n*)	77.8 (7)	72.4 (21)	100.0 (21)	83.1 (49)	0.03
Median pain score scale 1–10 (IQR)	2 (1.5–7)	5 (1–7)	6 (4–8.5)	5 (2–8)	0.05
Cervical prep after going home second day	*n* = 8	*n* = 26	*n* = 18	*n* = 52	
Reported pain, % (*n*)	62.5 (5)	69.2 (18)	100.0 (18)	78.8 (41)	0.02
Median pain score scale 1–10 (IQR)	2 (1–4)	4 (1–6.25)	7 (4–9)	4 (2–7)	0.003^c^
Digoxin administration^d^	*n* = 16	*n* = 40	*n* = 24	*n* = 24	
Reported pain, % (*n*)			87.5 (21)	87.5 (21)	N/A
Median pain score scale 1–10 (IQR)			3 (2–5)	3 (2–5)	N/A
Overall pain	*n* = 20	*n* = 41	*n* = 24	*n* = 85	
Reported pain, % (*n*)	85.0 (17)	78.0 (32)	100.0 (24)	85.9 (73)	0.049
Median pain score scale 1–10 (IQR)	5 (3–7.5)	5 (2–8)	6 (4–8)	5 (3–8)	0.43

Data presented as % (*n*) unless otherwise indicated.

^a^ Pearson chi square to compare proportions; Kruskal-Wallis to compare medians.

^b^ Dunn-Bonferroni adjusted pairwise comparison between16–17.6 and 18–20.6 weeks: *p* = 0.002.

^c^ Dunn-Bonferroni adjusted pairwise comparison between16–17.6 and ≥21 weeks: *p* = 0.005; between 18–20.6 and ≥21 weeks: *p* = 0.04.

^d^ During the data cleaning process, we identified that 5 respondents in the 16- to 17.6-week group and 40 respondents in the 18- to 20.6-week group rated pain from the digoxin injection, when clinic protocol does not indicate digoxin until gestational age is 21 weeks. We recoded responses for these five participants to reflect that they did not get digoxin per clinic protocol.

Median pain scores among the total sample did not exceed 5 (out of 10) at any of the time points during the abortion that were asked about on the survey. However, when disaggregated by gestational age, a median pain score of 7 was assessed in the 18.0- to 20.6-week group for cervical preparation in clinic on day 1 and in the 21- to 23.6-week cohort for cervical preparation after leaving the clinic on day 2. After day 1, the median pain score was significantly higher in the 18- to 20.6-week group versus 16- to 17.6-week group (5 vs. 3, *p* = 0.002) and after day 2, the median pain score was significantly higher in the 21- to 23.6-week group than in the 16- to 17.6-week group (7 vs. 2, *p* = 0.005) and the 18- to 20.6-week group (7 vs. 4, *p* = 0.04) (Table 2).

**FIG. 2.**
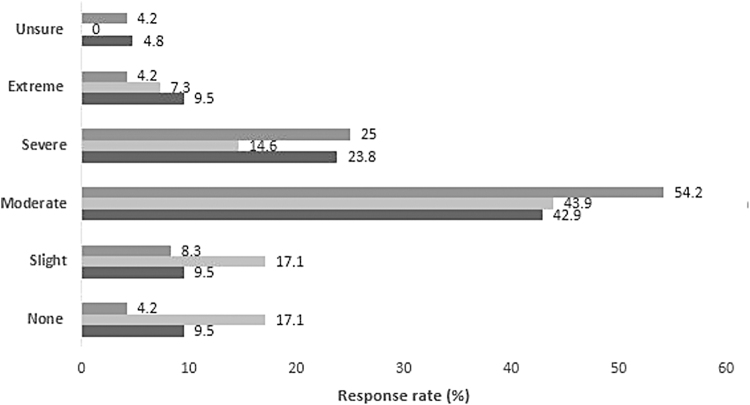
Client reported overall pain and discomfort with abortion procedure by gestational age (weeks) 
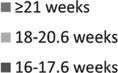
 *p*-value = 0.71; Pearson chi square to compare proportions.

When asked to qualitatively rate their pain, most participants reported moderate (47%) or severe (20%) overall pain and discomfort during the abortion procedure, with 7% reporting extreme pain and discomfort and 12% reporting no pain and discomfort. Characterization of pain in this way did not vary by gestational age group (Fig. 2).

Respondents most commonly reported that the pain experienced was less than or same as expected, however, more than one-quarter of the sample reported that it exceeded expectations. When broken down by gestational age group, it held that participants primarily felt that the pain experienced was less than what they expected (Table 2).

### Side effects

**FIG. 3.**
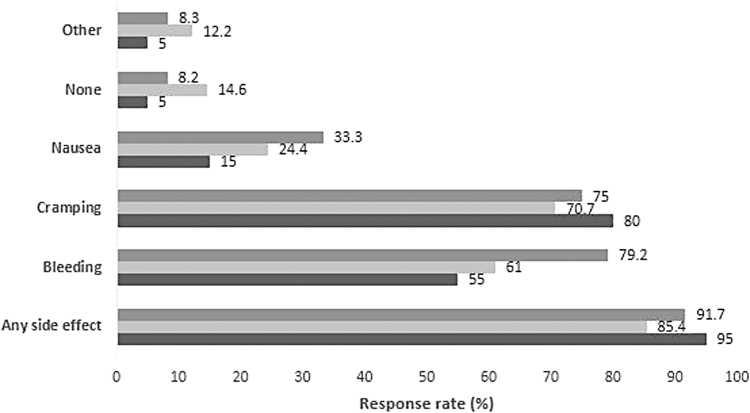
Client reported side effects from overall abortion experience by gestational age (weeks) 
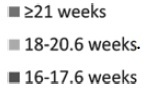
 *Other: 16–17.6 not specified; 18–20.6 feeling faint, fatigue/sleepiness, pressure; ≥21 diarrhea and water broke. All *p*-values >0.05; Fisher's exact test to compare proportions.

Nearly all (89%) respondents reported experiencing at least one side effect from the overall abortion experience, most commonly cramping (74%) and bleeding (65%). Approximately 11% of respondents reported no side effects (Fig. 3). Most participants reported that the side effects experienced were less than or the same as expected; approximately one-fifth reported they were more than expected. Reporting of side effects did not differ by gestational age (Table 3).

**Table 3.** Actual Versus Expected Pain, Side Effects, and Abortion Experience by Gestational Age (Weeks)

	16–17.6	18–20.6	≥21	Total	*p* ^a^
Pain	*n* = 21	*n* = 40	*n* = 24	*n* = 85	
More than expected	14.3 (3)	32.5 (13)	29.2 (7)	27.1 (23)	0.28
Same as expected	38.1 (8)	20.0 (8)	8.3 (2)	21.2 (18)	
Less than expected	42.9 (9)	45.0 (18)	58.3 (14)	48.2 (41)	
Unsure	4.8 (1)	2.5 (1)	4.2 (1)	3.5 (3)	
Side effects	*n* = 20	*n* = 40	*n* = 23	*n* = 84	
More than expected	10.0 (2)	22.0 (9)	30.4 (7)	21.4 (18)	0.39
Same as expected	50.0 (10)	31.7 (13)	21.7 (5)	33.3 (28)	
Less than expected	30.0 (6)	34.1 (14)	43.5 (10)	35.7 (30)	
Unsure	10.0 (2)	12.2 (5)	4.3 (1)	9.5 (8)	
Abortion experience	*n* = 20	*n* = 41	*n* = 23	*n* = 84	
Better than expected	65.0 (13)	82.0 (34)	78.3 (18)	77.4 (65)	0.16
Same as expected	30.0 (6)	4.9 (2)	17.4 (4)	14.3 (12)	
Worse than expected	0 (0)	4.9 (2)	4.3 (1)	3.6 (3)	
Unsure	5.0 (1)	7.3 (3)	0 (0)	4.8 (4)	

Data presented as % (*n*).

^a^ Pearson chi square to compare proportions.

### Acceptability

Respondents' assessments of their overall abortion experience were mostly positive. All respondents in the 21- to 23.6-week group rated their overall abortion experiences as positive or very positive, except for one person who was unsure. Only three participants rated their experiences as somewhat negative, and all were in the 18- to 20.6-week group (Table 3). More than three-fourths of respondents classified the abortion experience as better than expected, regardless of gestational age. Two people in the 18- to 20.6-week group and one person in the 21- to 23.6-week group reported that it was worse than expected (Table 3).

Shorter wait time was cited at a similar rate as less pain across gestational ages to improve patient experience. Fewer visits to the clinic was rarely reported as a way to improve experience, even in the group that more frequently required three visits (Table 4).

**Table 4.** Overall Experience of Abortion and Recommendations Regarding Pain and Number of Visits to Improve Experience by Gestational Age (Weeks)

	16–17.6	18–20.6	≥21	Total	*p* ^a^
Abortion experience	*n* = 20	*n* = 41	*n* = 24	*n* = 85	
Very positive	50.0 (10)	70.7 (29)	75.0 (18)	67.1 (57)	0.12
Somewhat positive	20.0 (4)	9.8 (4)	20.8 (5)	15.3 (13)	
Neutral	25.0 (5)	9.8 (4)	0 (0)	10.6 (9)	
Somewhat negative	0 (0)	7.3 (3)	0 (0)	3.5 (3)	
Negative	0 (0)	0 (0)	0 (0)	0 (0)	
Unsure	5.0 (1)	2.4 (1)	4.2 (1)	3.5 (3)	
Recommendations to improve experience	*n* = 19	*n* = 37	*n* = 23	*n* = 79	*p* ^b^
Less pain during cervical prep	15.8 (3)	29.7 (11)	47.8 (11)	31.6 (25)	0.08
Less pain during procedure	26.3 (5)	35.1 (13)	17.4 (4)	27.8 (22)	0.32
Less pain after procedure	0 (0)	5.4 (2)	13.0 (3)	6.3 (5)	0.21
Shorter wait times at clinic	57.9 (11)	35.1 (13)	30.4 (7)	39.2 (31)	0.15
Fewer visits to complete procedure	5.3 (1)	5.4 (2)	17.4 (4)	8.9 (7)	0.23

Data presented as % (*n*).

^a^ Pearson chi square to compare proportions.

^b^ Fisher's exact test to compare proportions.

## Discussion

Our findings support that longer duration of pregnancy at the time of abortion is associated with reporting higher pain scores with the procedure. Pregnancies at later gestational ages require more cervical dilation and larger uteruses may produce stronger contractions induced by misoprostol. It is not unexpected that pain was less frequent after day 1 for respondents with 16- to 17.6-week pregnancies, especially since 11/22 people in that cohort underwent quicker cervical preparation processes and likely completed their abortion procedures in 1 day.

Although pain frequency increased with gestational age, pain intensity was similar across gestational age cohorts. Reponses to the survey question about the severity of overall pain and discomfort (Fig. 2) suggested that pain was more severe than did the median pain scores, which underscores the challenges to researching, qualifying, and addressing pain, particularly since it is subjective, variable, and can be further heightened by anxiety.^25–27^

It is interesting to note that even though frequency of pain was higher in the 21- to 23.6-week cohort, report of a positive overall abortion experience was nonetheless common. It is not unusual in abortion studies to observe high rates of satisfaction and acceptability despite undesirable pain and side effects since participants have accessed needed care.^28^

It is not possible to determine from our data whether the clinic's pain management strategies effectively reduced pain, but our findings do suggest that there is a desire for additional pain relief strategies during the abortion procedure, regardless of gestational age. There is a dearth of research on pain management during later abortion—a recent review has noted that there is limited evidence on pain management strategies for abortion generally, and even a bigger lack of evidence for later abortion specifically.^26^

The World Health Organization (WHO),^29^ American College of Obstetrics and Gynecology,^30^ and the National Abortion Federation (NAF)^31^ identify various approaches to pain management, such as analgesia, anxiolytics, local anesthetic, conscious sedation, and general anesthesia, particularly after dilation is achieved, in their respective clinical guidance, but none provides evidence-based recommendations for specific protocols especially for less resource intensive approaches, which might be preferred and are important for clinics without the infrastructure for intravenous sedation. Less complicated and more accessible pain management strategies should be prioritized for future research.

We did not find any association between side effects and gestational age, which is not necessarily unexpected. Misoprostol was used for cervical preparation in all gestational age groups and its known side effects, particularly cramping and nausea, were consistently reported. The routine use of digoxin at 18 weeks and beyond could have increased the frequency of nausea, but the group sizes are small and it is possible that additional doses of misoprostol in these later cohorts contributed to reports of nausea; we cannot separate out the effect of digoxin on nausea.

Survey respondents with pregnancies in the highest gestational age group were more likely to be pregnant for the first time and to be nulliparous compared to those in the lowest gestational age group. The published literature reflects that primary delays to care include difficulty recognizing or confirming pregnancy.^32–34^

In our survey sample, participants in the 21- to 23.6-week group were not younger than those in the other gestational age groups even though delays in pregnancy recognition can be more acute at younger ages.^33,35^

Other research have reported that people with children are also more likely to take longer to get an abortion, possibly due to arranging childcare and making appointments around familial commitments;^32^ however, there was no statistically significant difference in the proportions of survey respondents with children when grouped by gestational age.

Our study has a few limitations. Stratifying results by cervical preparation procedure protocol was not meaningful because group sizes were so small. Another limitation is that gestational age was based on self-report and was not confirmed with clinic records. We did identify some gestational age-related inconsistencies before data cleaning, such as respondents in the earliest gestational age cohort who reported receiving digoxin when neither the original or modified cervical preparation protocols called for it, but it is unlikely that any gestational age reporting errors were widespread, as the clinic uses ultrasound to date gestational age and provides patients with the gestational age of the pregnancy before patient education.

In addition, it is possible that respondents misunderstood the lowest rating of the visual analog pain scale, where one was meant to indicate no pain and corresponded to a small circle that could have been misconstrued to represent minimal pain. Finally, statistical analyses conducted on these small group sizes cannot be generalized to the larger population.

## Conclusions

In this study, people seeking abortion care at later gestations in the second trimester were more likely to report pain during their abortions. Although most respondents were prepared for the pain they experienced, some reported experiencing more pain than they expected, and more effective pain relief was commonly reported as a way to improve the service.

The findings suggest both that the clinic staff could provide more information about expected pain during counseling and that more research is needed to establish evidence-based pain management strategies, especially for cervical preparation. As pain generally increases with gestational age, future research should be powered to assess outcomes by gestational age to yield more concrete conclusions about differences in pain, side effects, and acceptability.

## Abbreviations Used

D&E: dilation and evacuation
IQR: interquartile range
